# Different DNA End Configurations Dictate Which NHEJ Components Are Most Important for Joining Efficiency*[Fn FN2]

**DOI:** 10.1074/jbc.M116.752329

**Published:** 2016-10-04

**Authors:** Howard H. Y. Chang, Go Watanabe, Christina A. Gerodimos, Takashi Ochi, Tom L. Blundell, Stephen P. Jackson, Michael R. Lieber

**Affiliations:** From the ‡ Departments of Pathology, Biochemistry & Molecular Biology, and Molecular Microbiology & Immunology and the Section of Molecular & Computational Biology, Department of Biological Sciences, Norris Comprehensive Cancer Center, University of Southern California Keck School of Medicine, Los Angeles, CA, 90033 and; the § Gurdon Institute and Department of Biochemistry, University of Cambridge, Cambridge CB2 1QN, United Kingdom

**Keywords:** chromosomes, DNA damage, DNA recombination, DNA repair, enzyme, double-strand DNA break, ligase, nuclease

## Abstract

The nonhomologous DNA end-joining (NHEJ) pathway is a key mechanism for repairing dsDNA breaks that occur often in eukaryotic cells. In the simplest model, these breaks are first recognized by Ku, which then interacts with other NHEJ proteins to improve their affinity at DNA ends. These include DNA-PK_cs_ and Artemis for trimming the DNA ends; DNA polymerase μ and λ to add nucleotides; and the DNA ligase IV complex to ligate the ends with the additional factors, XRCC4 (X-ray repair cross-complementing protein 4), XLF (XRCC4-like factor/Cernunos), and PAXX (paralog of XRCC4 and XLF). *In vivo* studies have demonstrated the degrees of importance of these NHEJ proteins in the mechanism of repair of dsDNA breaks, but interpretations can be confounded by other cellular processes. *In vitro* studies with NHEJ proteins have been performed to evaluate the nucleolytic resection, polymerization, and ligation steps, but a complete system has been elusive. Here we have developed a NHEJ reconstitution system that includes the nuclease, polymerase, and ligase components to evaluate relative NHEJ efficiency and analyze ligated junctional sequences for various types of DNA ends, including blunt, 5′ overhangs, and 3′ overhangs. We find that different dsDNA end structures have differential dependence on these enzymatic components. The dependence of some end joining on only Ku and XRCC4·DNA ligase IV allows us to formulate a physical model that incorporates nuclease and polymerase components as needed.

## Introduction

The mammalian genome is a vast target for genotoxic agents. It is estimated that the genome undergoes ∼100,000 alterations/day, which can result in an estimated 10 double-stranded breaks (DSBs)/day (1–5).^3^ The NHEJ DNA repair pathway is required to repair many of these DSBs.

The NHEJ pathway first begins with the toroid-shaped Ku heterodimer (Ku70 and Ku80) binding to the free dsDNA ends. Ku then recruits other NHEJ factors as needed (6).

Many components improve NHEJ efficiency *in vivo*, but the only essential component for NHEJ is the DNA ligase complex consisting of XRCC4·DNA ligase IV (X4·LIV) in a 2:1 ratio that facilitates the ligation of a 3′-hydroxyl to a 5′-phosphate at the partner DNA end (7–9). More recently, NHEJ accessory factors, XLF and PAXX, have been discovered that bind to DNA ligase IV and Ku, respectively (10–14). Both XLF and PAXX have been shown to promote the ligation of DNA ends that do not have any terminal base pairing or microhomology (incompatible ends) (10–12, 14). However, if the DNA ends require processing (*e.g.* if they are incompatible), a nuclease or polymerase may become essential. Artemis appears to be the major NHEJ nuclease (15), although other nucleases, such as APLF (also called PALF), may participate in limited cases, especially when Artemis is not present (16). Artemis has 5′ exonuclease activity by itself and acquires endonuclease activity when complexed with autophosphorylated DNA-PK_cs_ (15, 17).

**FIGURE 1.**
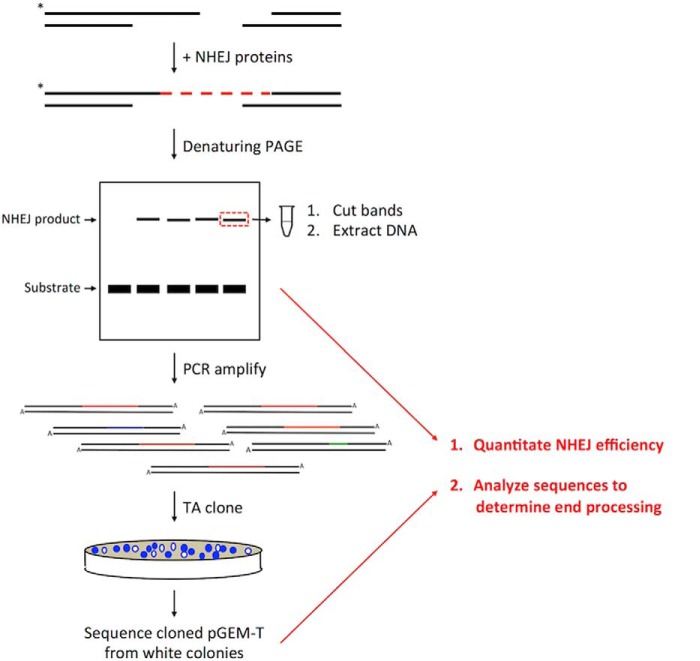
**NHEJ reconstitution workflow.** NHEJ proteins are added to labeled DNA substrate, and NHEJ products are resolved by denaturing PAGE. NHEJ products are cut out and PCR-amplified. PCR products are TA cloned into a pGEM-T vector and transformed into *E. coli*. Vector is then isolated from colonies, and the junctions are sequenced.

A NHEJ reconstitution system in which we can observe the joined products directly following PAGE would provide information on the ligation of each strand of the duplex, allow us to determine NHEJ efficiencies, and provide detailed mechanistic insight after sequencing the junctions (Fig. 1). One earlier direct gel NHEJ reconstitution did not include a nuclease but rather focused on the ability of the ligase complex to ligate across gaps in either strand and the ability of polymerase μ and λ to add random nucleotides (nt), thereby providing new microhomology (MH) between two DNA ends (18). Another early reconstitution included all known nuclease, polymerase, and ligase components but was not sufficiently efficient to permit direct gel assessment of joining products and tested only one pair of DNA end configurations (19). None of the reconstitutions have demonstrated a role for DNA-PK_cs_. The study here is the first direct gel NHEJ reconstitution system that includes all major nuclease, polymerase, and ligase components and DNA-PK_cs_. We find that the contribution of each major component to the efficiency of DNA end joining depends on the configuration of the two DNA ends being joined in a manner that permits a coherent model for NHEJ to be proposed.

## Results

### 

#### 

##### Resection-dependent Compatible DNA Ends Require Artemis and Are Strongly Stimulated by Ku and DNA-PK_cs_

**FIGURE 2.**
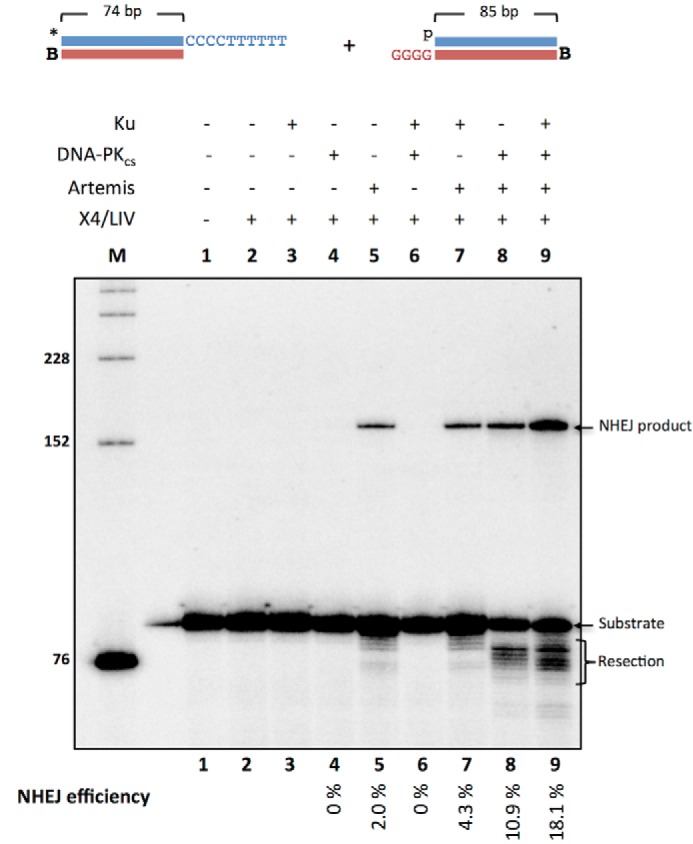
**NHEJ of resection-dependent compatible 3′ overhang requires Artemis and is strongly stimulated by Ku and DNA-PK_cs_.** NHEJ proteins (50 nm Ku, 25 nm DNA-PK_cs_, 25 nm Artemis, 100 nm X4·LIV) were incubated for 60 min at 37 °C with 20 nm *HC101/102 and 20 nm pHC115/116 in a reaction buffer containing 200 nm streptavidin to bind to biotin (*B*) to block one end of the DNA. In addition, *p* represents a 5′ phosphate, and the *asterisk* represents the radiolabel. NHEJ efficiencies are noted underneath. The reported values are averages of three independent experiments with a S.E. of 0.17% (*lane 5*), 1.0% (*lane 7*), 2.1% (*lane 8*), and 4.4% (*lane 9*).

Ligation of DNA ends with MH of even 1 base pair can be more efficiently ligated by the X4·LIV complex than blunt DNA ends. We wondered whether DNA ends with 3′ overhangs without terminal MH but with internal MH would undergo ligation. We incubated a 5′-radiolabeled DNA substrate with a (CCC CTT TTT T-3′) overhang and a substrate with a (GGG G-3′) overhang with Ku, DNA-PK_cs_, Artemis, and X4·LIV, as indicated (Fig. 2). These two DNA ends have the potential of generating 1–4-bp MH. The 3′ end of the bottom strand of the labeled duplex and the 5′ end of the bottom strand of the unlabeled duplex were biotinylated so that we could block one end of each duplex by adding streptavidin. The string of Ts in the 3′ overhang is intended to prevent base pairing until the Ts are resected. In this paper, we use the phrase “resection-dependent” to refer to joining that depends on any loss of nucleotides, regardless of DNA strand polarity. Supporting this point, we observed that NHEJ ligation products are not generated unless the reactions contain the Artemis nuclease (Fig. 2, *lanes 2–4* and *lane 6*).

However, pairwise or more complete reactions have measurable ligation. Approximately 2% of the substrate is converted to ligated product when Artemis and X4·LIV are present (Fig. 2, *lane 5*). The addition of Ku does not change NHEJ efficiency (Fig. 2, *lane 7*). When Artemis and DNA-PK_cs_ are used along with X4·LIV, NHEJ efficiency increases to 11% (Fig. 2, *lane 8*). The addition of Ku to DNA-PK_cs_, Artemis, and X4·LIV further increases NHEJ efficiency to 18% (Fig. 2, *lane 9*). We describe this event as resection-dependent (RD)-compatible end NHEJ because ligation can occur efficiently if the Artemis·DNA-PK_cs_ complex is present to efficiently remove the nonhomologous portion of the overhang so that the region of MH can be used to stabilize the DNA ends for ligation.

##### X4·LIV Stimulates DNA-PK_cs_-independent Artemis Activity

**FIGURE 3.**
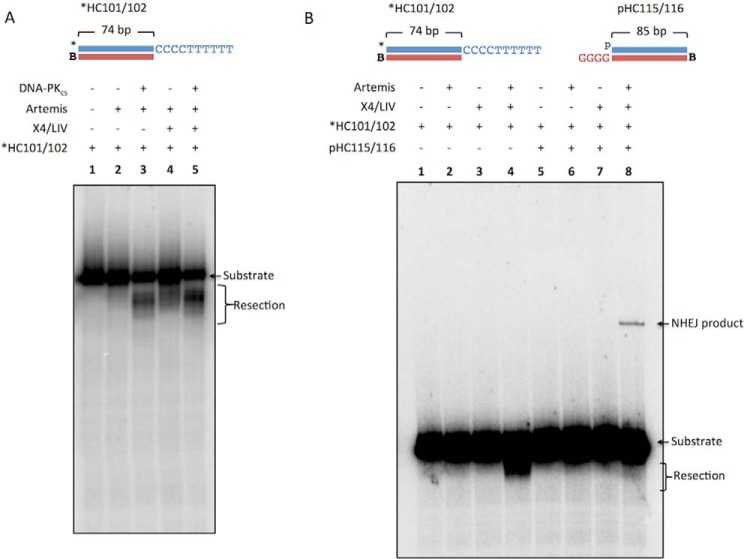
**Artemis resection of 3′ overhangs is stimulated by X4·LIV.** NHEJ proteins were incubated for 60 min at 37 °C with a reaction buffer containing 200 nm streptavidin to bind to biotin (*B*) to block one end of the DNA. In addition, *p* represents a 5′ phosphate, and the *asterisk* represents the radiolabel. *A*, 20 nm *HC101/102 was incubated with 50 nm Artemis (*lane 2*); 25 nm DNA-PK_cs_ and 50 nm Artemis (*lane 3*); 50 nm Artemis and 100 nm X4·LIV (*lane 4*); and 25 nm DNA-PK_cs_, 50 nm Artemis, and 100 nm X4·LIV (*lane 5*). *B*, *20 nm HC 101/102 was incubated with 20 nm pHC115/116 and 50 nm Ku, 25 nm DNA-PK_cs_, 25 nm Artemis, and 100 nm X4·LIV as indicated.

We were curious why there was resection and NHEJ products in Artemis and X4·LIV conditions without DNA-PK_cs_ (Fig. 2, *lane 5*). Artemis has intrinsic 5′ exonuclease activity and endonuclease activity when complexed with DNA-PK_cs_. We incubated the (CCC CTT TTT T-3′) overhang substrate with either Artemis alone; DNA-PK_cs_ and Artemis; Artemis and X4·LIV; or DNA-PK_cs_, Artemis, and X4·LIV (Fig. 3*A*). It has been reported that the C-terminal region of Artemis (amino acids 485–495) may interact with the DNA binding domain of DNA ligase IV (20, 21). We find that Artemis resection of the 3′ overhang increases when X4·LIV is added (Fig. 3*A*, *lane 2 versus lane 4*). We next tested whether the Artemis resection requires the presence of a partner substrate that may be able to generate a 3′ flap if the two overhangs transiently anneal by forming C-G base pairs prior to resection of the dTs (Fig. 3*B*). We find that Artemis resection of the 3′ overhang is stimulated by the addition of X4·LIV, independent of a second partner substrate (Fig. 3*B*, *lane 4*).

##### Resection-dependent Compatible Ends Rely on MH for NHEJ

**FIGURE 4.**
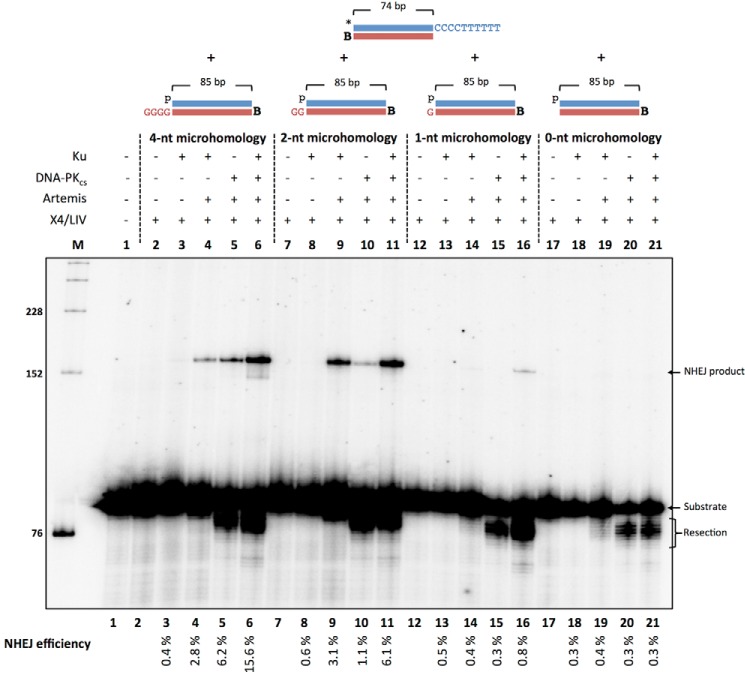
**NHEJ of resection-dependent compatible 3′ overhangs is strongly dependent on microhomology.** NHEJ proteins (50 nm Ku, 25 nm DNA-PK_cs_, 25 nm Artemis, 100 nm X4·LIV) were incubated with 20 nm *HC101/102 and either 20 nm pHC115/116 (*lanes 1- 6*), pHC115/123 (*lanes 7- 11*), pHC115/124 (*lanes 12- 16*), or pHC115/120 (*lanes 17- 21*) for 60 min at 37 °C in a reaction containing 200 nm streptavidin to bind to biotin (*B*) to block one end of the DNA. In addition, *p* represents a 5′ phosphate, and the *asterisk* represents the radiolabel. NHEJ efficiencies are noted underneath. The S.E. values from independent experiments are 0.05% (*lane 3*), 0.55% (*lane 4*), 1.75% (*lane 5*), 0.4% (*lane 6*), 0.05% (*lane 8*), 0.1% (*lane 9*), 0.35% (*lane 10*), 1.45% (*lane 11*), 0.1% (*lane 13*), 0.1% (*lane 14*), 0.05% (*lane 15*), 0.15% (*lane 16*), 0.05% (*lane 18*), 0.05% (*lane 19*), 0.05% (*lane 20*), and 0.05% (*lane 21*). Variations in total recovery are less marked in replicates of the same experiment, and the conversion of substrate to product is unaffected by variations in total recovery.

We next wondered whether the NHEJ of RD-compatible ends utilized the MH generated when the 3′ Ts in the overhang (CCC CTT TTT T-3′) are resected by the Artemis·DNA-PK_cs_ complex. We incubated NHEJ proteins and the 3′ overhang substrate (CCC CTT TTT T 3′) with a partner 3′ overhang substrate that has the potential to generate 1–4-bp MH in the overhangs (GGGG-3′) (Fig. 4, *lanes 2–6*), 1–2-bp MH (GG-3′) (Fig. 4, *lanes 7–11*), 1-bp MH (G-3′) (Fig. 4, *lanes 12–16*), or 0-bp MH (Fig. 4, *lanes 17–21*). NHEJ efficiency decreases as the MH decreased from 16% with 4-bp MH, 6% with 2-bp MH, 1% with 1-bp MH, and <1% with 0-bp MH (Fig. 4, *lane 6 versus lane 11 versus lane 16 versus lane 21*). These data suggest that NHEJ of RD-compatible ends utilizes the Artemis·DNA-PK_cs_ complex activity to resect the overhang to produce a region of MH for ligation to occur.

##### Incompatible 3′ Ends Cannot be Joined in a Biochemical System Containing Ku, DNA-PK_cs_, Artemis, and X4·LIV Complex Alone

**FIGURE 5.**
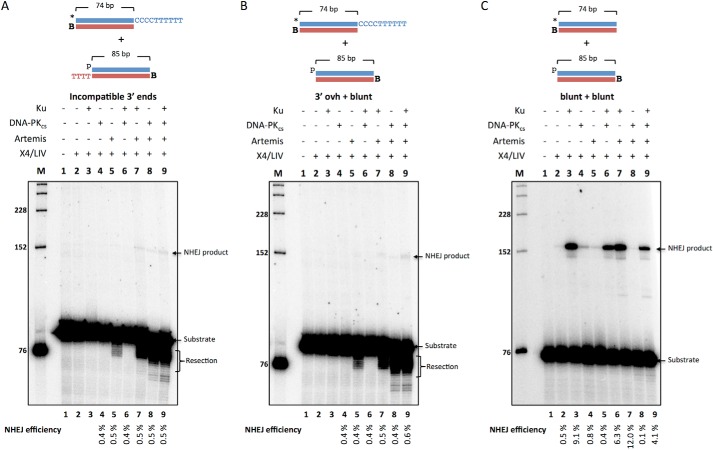
**The Ku, DNA-PK_cs_, Artemis, and X4·LIV complex is not sufficient for NHEJ of incompatible DNA ends, whereas blunt-ended DNA only requires Ku and X4·LIV.** NHEJ proteins (50 nm Ku, 25 nm DNA-PK_cs_, 25 nm Artemis, 100 nm X4·LIV) were incubated for 60 min at 37 °C in a reaction buffer containing 200 nm streptavidin to bind to biotin (*B*) to block one end of the DNA. In addition, *p* represents a 5′ phosphate, and the *asterisk* represents the radiolabel. NHEJ efficiencies are noted underneath. *A*, DNA substrates used were 20 nm *HC101/102 and 20 nm pHC115/119. *B*, DNA substrates used were 20 nm *HC101/102 and 20 nm pHC115/120. *C*, DNA substrates used were 20 nm *HC121/102 and 20 nm pHC115/120. These are representative gels of three similar experiments that have confirmed these results.

We next attempted to increase the complexity of *in vitro* NHEJ by testing substrates with no regions of MH in the overhangs (*i.e.* incompatible ends). Joining of a DNA end with a (CCC CTT TTT T-3′) overhang to an end with a (TTT T-3′) overhang showed marginal levels of NHEJ (Fig. 5*A*, *lanes 7–9*). Similarly, NHEJ of a DNA end with a (CCC CTT TTT T-3′) overhang with a blunt-ended DNA end pair showed marginal levels of NHEJ (Fig. 5*B*, *lanes 7–9*). Because of the small percentage of total signal, the visible but faint NHEJ product bands in Fig. 5 (*A* and *B*, *lanes 7–9*) were not quantitatively greater than the gel background. These data suggest that Artemis·DNA-PK_cs_ resection activity alone is not sufficient to promote NHEJ of fully incompatible DNA ends. In contrast, blunt end ligation required only Ku and X4·LIV (Fig. 5*C*, *lane 3*). The addition of DNA-PK_cs_ actually inhibited ligation ∼2-fold irrespective of whether Artemis is also included (Fig. 5*C*, *lane 3 versus lane 6 versus lane 7 versus lane 9*). These data suggest that NHEJ of blunt-ended substrates in our biochemical system proceeds by direct ligation without any requirement for MH or terminal base pairing after resection.

##### Pol μ but Not Pol λ Stimulates NHEJ of 3′ Incompatible DNA Ends

**FIGURE 6.**
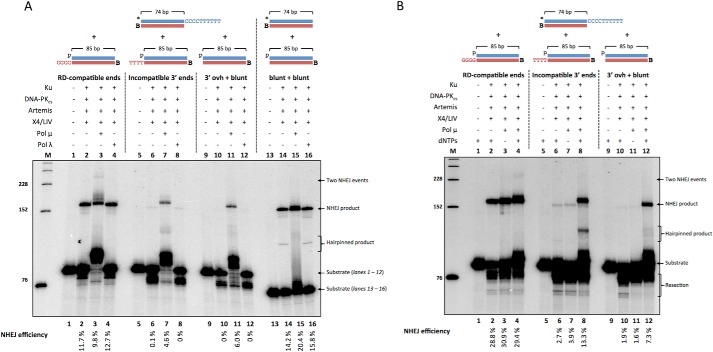
**NHEJ of 3′ incompatible ends is stimulated by Pol μ but not Pol λ.** NHEJ proteins (50 nm Ku, 25 nm DNA-PK_cs_, 25 nm Artemis, 100 nm X4·LIV, 25 nm Pol μ, and 25 nm Pol λ) were incubated for 60 min at 37 °C in a reaction containing 200 nm streptavidin to bind to biotin (*B*) to block one end of the DNA. In addition, *p* represents a 5′ phosphate, and the *asterisk* represents the radiolabel. NHEJ efficiencies are noted underneath. *A*, DNA substrates used were 20 nm *HC101/102 and either 20 nm pHC115/116 (*lanes 1–4*), pHC115/119 (*lanes 5–8*), or pHC115/120 (*lanes 9–12*). Blunt end ligations were performed with 20 nm *HC121/102 and 20 nm pHC115/120 (*lanes 13–16*). *B*, 100 μm dNTPs were incubated with DNA substrates 20 nm *HC101/102 and either 20 nm pHC115/116 (*lanes 1–4*), pHC115/119 (*lanes 5–8*), or pHC115/120 (*lanes 9–12*). These are representative gels of multiple similar experiments that have confirmed these results.

Because Ku, Artemis, DNA-PK_cs_, and X4·LIV were not sufficient for NHEJ of incompatible 3′ overhangs, we tested whether Pol X family polymerases can stimulate NHEJ. The Pol X family members consist of Pol μ, Pol λ, Pol β, and terminal deoxynucleotidyl transferase (TdT). Pol β and TdT do not participate in NHEJ, except for the role of TdT in NHEJ in pre-B or pre-T cells (4). Pol μ has the ability to add nucleotides in either a template-dependent or template-independent manner (22). Conversely, Pol λ is primarily a template-dependent polymerase, with much less template-independent activity (22). The addition of Pol μ or Pol λ did not significantly increase NHEJ of RD-compatible ends (Fig. 6, A, *lanes 2–4*, and *B*, *lane 2 versus lane 4*). However, Pol μ was able to promote two sequential ligation events, suggesting that streptavidin does not completely block one end from ligation and that Pol μ may be promoting NHEJ (Fig. 6, *A*, *lane 3*, and *B*, *lane 4*). These data suggest that resection, and not nucleotide addition, are sufficient for NHEJ of RD-compatible DNA ends, but Pol μ may be able to add nt that would generate occult MH (4).

NHEJ of 3′ incompatible DNA ends increased from ∼0.1 to 4.6% with the addition of Pol μ but not Pol λ (Fig. 6*A*, *lanes 6–8*). A similar increase from undetectable to 6.0% was observed with the addition of Pol μ in the NHEJ of a 3′ overhang with blunt-ended DNA partner (Fig. 6*A*, *lanes 10–12*). Furthermore, Pol μ does not provide a DNA end stabilization function, as indicated by the lack of NHEJ improvement upon Pol μ inclusion in dNTP-free conditions (Fig. 6*B*, *lanes 2–4*, *6–8*, and *10–12*). (Weaker intensity bands that migrated between the substrate and NHEJ product are likely DNA hairpinned products from NHEJ of two molecules of the identical duplex followed by melting and intramolecular annealing of each strand (18).) These data suggest that Pol μ may be adding nucleotides to help form regions of MH for NHEJ of substrates with incompatible 3′ overhangs. Because Pol λ does not stimulate NHEJ, the contribution of Pol μ most likely proceeds via its template-independent addition.

A smaller effect was observed in the NHEJ of blunt-ended DNA. Pol μ only marginally increased NHEJ from 14.2% to 20.4%, whereas Pol λ did not change NHEJ (Fig. 6*A*, *lanes 14–16*). These data support our other findings suggesting that blunt-ended NHEJ may proceed through direct ligation even though the Artemis·DNA-PK_cs_ complex, which is capable of resecting nucleotides from the blunt ends, is present.

##### PAXX, but Not XLF, Stimulates NHEJ of Blunt DNA Ends

**FIGURE 7.**
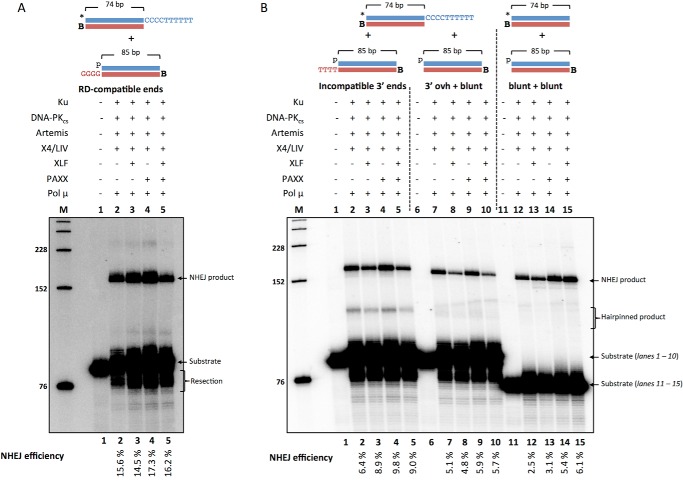
**PAXX only stimulates blunt end NHEJ.** NHEJ proteins (50 nm Ku, 25 nm DNA-PK_cs_, 25 nm Artemis, 100 nm X4·LIV, 500 nm PAXX, 200 nm XLF, and 25 nm Pol μ) were incubated for 60 min at 37 °C in a reaction containing 200 nm streptavidin to bind to biotin (*B*) to block one end of the DNA. In addition, *p* represents a 5′ phosphate, and the *asterisk* represents the radiolabel. *A*, RD-compatible NHEJ was performed with 20 nm *HC101/102 and 20 nm pHC115/116. NHEJ efficiencies are noted underneath. The reported values are averages of three independent experiments with a S.E. of 0.1% (*lane 2*), 0.2% (*lane 3*), 0.6% (*lane 4*), and 0.4% (*lane 5*). *B*, DNA substrates used were 20 nm *HC101/102 and either 20 nm pHC115/119 (*lanes 1–5*) and pHC115/120 (*lanes 6–10*). Blunt end ligations were performed with 20 nm *HC121/102 and 20 nm pHC115/120 (*lanes 11–15*). NHEJ efficiencies are noted underneath. The reported values are averages of three independent experiments with a S.E. of 0.6% (*lane 2*), 0.3% (*lane 3*), 0.4% (*lane 4*), 0.6% (*lane 5*), 0.3% (*lane 7*), 0.3% (*lane 8*), 0.4% (*lane 9*), 0.2% (*lane 10*), 0.3% (*lane 12*), 0.4% (*lane 13*), 0.6% (*lane 14*), and 0.3% (*lane 15*).

We next wondered whether XLF and PAXX would improve joining in our biochemical NHEJ system. We incubated XLF and/or PAXX with Ku, DNA-PK_cs_, Artemis, X4·LIV, and Pol μ (Fig. 7). We observed that XLF and PAXX have no stimulatory effect on the NHEJ of RD-compatible ends (Fig. 7*A*, *lanes 2–5*). It is possible that at RD-compatible ends, other DNA ligase factors may be unnecessary.

NHEJ of incompatible 3′ ends was not improved with XLF or PAXX (Fig. 7*B*, *lanes 2–5*). The same was observed for the NHEJ of DNA with a 3′ overhang with a blunt-ended DNA partner (Fig. 7*B*, *lanes 7–10*). However, NHEJ of blunt-ended DNA was stimulated ∼2-fold by PAXX but not XLF (Fig. 7*B*, *lanes 12–15*). We had already reported the finding that XLF does not improve blunt-end ligation (23). These new data suggest that the role of PAXX may be to help align the 5′-phosphate and 3′-hydroxyl by interacting with Ku so that the ligase can ligate efficiently.

##### NHEJ of a 5′ Overhang to a Blunt End Is Stimulated by XLF and PAXX

**FIGURE 8.**
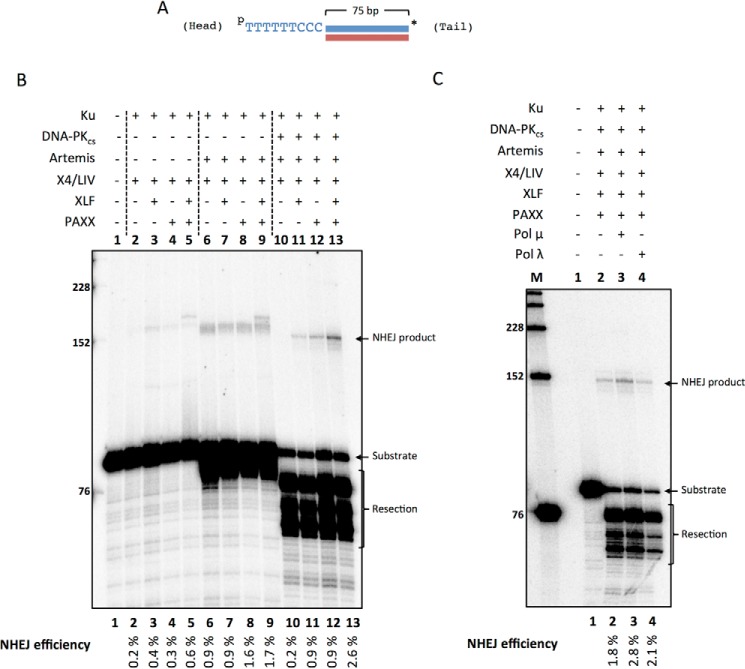
**NHEJ of 5′ overhang to a blunt end is stimulated by XLF and PAXX with no polymerase effect.** NHEJ proteins included 50 nm Ku, 25 nm DNA-PK_cs_, 25 nm Artemis, 100 nm X4·LIV, 500 nm PAXX, 200 nm XLF. *A*, depiction of DNA substrate where *p* represents a 5′ phosphate, and the *asterisk* represents the radiolabel. Pol μ and Pol λ were incubated with 20 nm pJG277*/JG226-ddG for 60 min at 37 °C. NHEJ efficiencies are noted underneath. *(Head)* refers to the end with the 5′ overhang, and *(Tail)* refers to the blunt end. *B*, XLF and PAXX were varied in NHEJ reactions with Ku and X4·LIV (*lanes 2–5*); Ku, Artemis, and X4·LIV (*lanes 6–9*); and Ku, DNA-PK_cs_, Artemis, and X4·LIV (*lanes 10–13*). *C*, Pol μ and Pol λ (25 nm each) were included in NHEJ reactions with Ku, DNA-PK_cs_, Artemis, X4·LIV, XLF, and PAXX as noted. These are representative gels of multiple similar experiments that have confirmed these results.

Next we tested the effect of XLF and PAXX on the NHEJ of a 5′ overhang substrate with the radiolabel on the blunt-ended 3′ end (Fig. 8*A*). This substrate can undergo head to tail, head to head, or tail to tail ligation events; however, both the head to head and tail to tail events will quickly form hairpins. The hairpinned DNA will migrate much faster than the linear products formed from head to tail events. Because we did not observe any hairpinned bands between the NHEJ product and substrate bands, we assumed that the preferred NHEJ event was the (5′-TTT TTT CCC) head ligating to the blunt-ended tail end of the substrate (Fig. 8). XLF and PAXX marginally improved the low levels of NHEJ observed in Ku and X4·LIV reactions (Fig. 8*B*, *lanes 2–5*). In reactions with Ku, Artemis, and X4·LIV, XLF and PAXX did not improve NHEJ alone, but marginally improved NHEJ with XLF and PAXX together (Fig. 8*B*, *lanes 6–9*). However, NHEJ was stimulated individually by XLF and PAXX in reactions containing Ku, DNA-PK_cs_, Artemis, and X4·LIV (Fig. 8*B*, *lanes 10–12*). The addition of both XLF and PAXX together increases NHEJ ∼3-fold from 1% (XLF or PAXX alone) to 3% (XLF and PAXX) (Fig. 8*B*, *lanes 11–13*). These data suggest that XLF and PAXX are able to promote NHEJ of a 5′ overhang with a blunt-ended DNA partner. Because the ligation product band migrates approximately at the 152-nt position, it is likely that the 5′ overhang is mostly processed prior to ligation (Fig. 8*B*, *lane 13*). In addition, the NHEJ products are larger when Artemis is not present (Fig. 8*B*, *lanes 3–5*) or when Artemis 5′ exonuclease activity is dominant in DNA-PK_cs_-free conditions (Fig. 8*B*, *lanes 6–9*).

We next tested the role of Pol μ and Pol λ in the NHEJ of the 5′ overhang to a blunt end. Both Pol μ and Pol λ only marginally stimulated NHEJ (Fig. 8*C*, *lanes 2–4*). These data suggest that the addition of nucleotides is not used to join DNA with blunt ends and 5′ overhangs.

##### NHEJ of RD-compatible Ends Reveals a Preference for Resection to Generate MH for Ligation

We next sequenced the junctions from the joining products by cutting out bands from the PAGE gels, PCR amplifying, and TA cloning them into a pGEM-T vector for sequencing (Fig. 1). We found that NHEJ of RD-compatible ends is maximal in Ku, DNA-PK_cs_, and Artemis conditions (Figs. 2, *lane 9*; 5*A*, *lane 2*; and 6*B*, *lane 2*). The sequences from these junctions showed that the preferred modification is to resect the 3′ dTs from the (CCC CCT TTT TT-3′) overhang to create ∼3–4-nt MH to stabilize ligation (Tables 1 and 2). Surprisingly, the addition of Pol μ did not result in any sequences with additional nucleotides (Table 1). In all junctions analyzed (*n* = 99), irrespective of NHEJ proteins involved, the end processing never proceeded into the duplex portion but rather stopped at or before the 3′ overhang (supplemental Table S1).

**TABLE 1:**
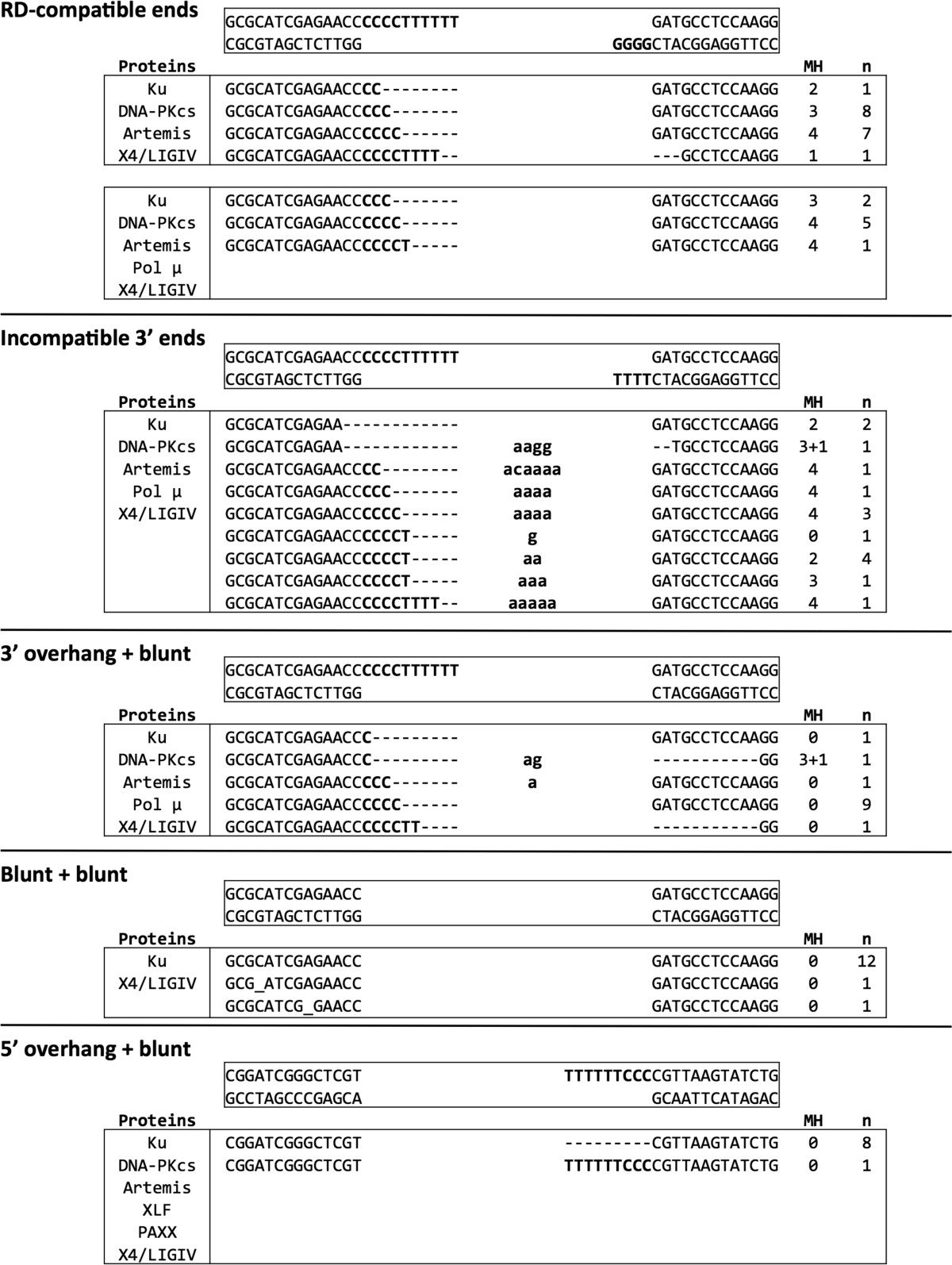
**Sequence results of the most efficient NHEJ events** Sequencing results of the NHEJ junctions with a column for proteins included, sequenced junctions, microhomology (MH) utilized at the junction, and the number of molecules sequenced (*n*). Dashes (–) represent resected bases, underscores (_) represent deletions, and bolded lowercase letters represent added bases. Only the top strand is shown.

**TABLE 2:** **NHEJ summary** The table describes the observed parameters for NHEJ of RD-compatible ends, 3′ incompatible overhangs, 3′ overhang with a blunt-ended partner, blunt-ended DNA, and 5′ overhang to a blunt end.

	Artemis-dependent NHEJ	Polymerase μ stimulation	Polymerase λ stimulation	XLF and PAXX stimulation	Preferred modifications
RD-compatible ends	++	−	−	−	Resection to expose MH
3′ incompatible ends	++	++	−	−	Resection; addition by Pol μ to create MH
3′ overhang + blunt	++	++	−	−	Resection; addition by Pol μ
Blunt + blunt	−	−	−	+ (PAXX)	Direct ligation
5′ overhang + blunt	++	−	−	+ (XLF and PAXX)	Resection of 5′ overhang; ligation without MH

##### NHEJ Junctions from the Joining of 3′ Incompatible Ends Reveal a Preference for Resection and Nucleotide Addition to Provide MH for Ligation

On the other hand, NHEJ of DNA ends with no potential microhomology on the 3′ overhangs (incompatible 3′ ends) was very weak without Pol μ (Fig. 6, *A*, *lane 7*, and *B*, *lane 8*). Hence, the predominant products show resection of nucleotides from the (CCC CCT TTT TT-3′) overhang and Pol μ synthesis of primarily dAs which creates 2- to 4-nt MH with the 3′ (TTT T-3′) on the other DNA end (Tables 1 and 2). When Pol μ was not included in the reactions, the preferred junctions involved complete resection of the (CCC CCT TTT TT-3′) overhang until the stretch of two dAs were exposed 3 bps into the duplex (supplemental Table S2). These dAs were then used to provide MH with the (TTT T-3′) overhang on the other DNA end. One unique sequence was the result of 3-bp MH and a ligation over a G-T mismatch (Table 1). Not surprisingly, X4·LIV was able to ligate over this mismatch (18).

##### NHEJ of a 3′ Overhang with a Blunt-ended Partner Reveals a Preference for Resection and Ligation without MH

We next examined the junction sequences from the NHEJ of a 3′ overhang ligated with a blunt-ended partner. NHEJ efficiency was best in reactions that contained Pol μ (Fig. 6, *A*, *lane 11*, and *B*, *lane 12*). Sequences of these junctions revealed that the DNA ends undergo resection on both ends of the DNA without significant nucleotide addition. Surprisingly, only 2 of the 13 sequenced junctions showed the addition of nucleotides (Table 1). Furthermore, only one of those sequences resulted from a 3-bp MH with ligation over a G-T mismatch (Table 1). Overall, the sequencing results indicate that the preferred repair process is to resect the overhang and resect into the duplex without the use of MH (Table 2).

##### NHEJ of Blunt-ended DNA Reveals Preference for Direct Ligation

NHEJ of blunt-ended DNA is a highly efficient process that only requires Ku and X4·LIV for maximal activity (Fig. 5*C*, *lane 3*). The addition of other NHEJ factors does not improve NHEJ and does not increase the diversity of junctions generated (supplemental Table S4). Thus, the preferred joining product from two blunt DNA ends is direct ligation (Tables 1 and 2).

##### NHEJ of a 5′ Overhang to a Blunt End Reveals Preference for Resection and Direct Ligation

NHEJ of a 5′ overhang to a blunt end is maximal in Ku, DNA-PK_cs_, Artemis, X4·LIV, XLF, and PAXX conditions (Fig. 8*B*, *lane 13*). Ensuing sequence analysis shows that the 5′ overhang is completely resected in most joints without processing of the blunt-ended partner (Table 1). Ligation then occurs without utilizing MH (Tables 1 and 2). In fact, sequencing of the Ku, Artemis, and X4·LIV condition also shows that ligation without MH is preferred (supplemental Table S5).

## Discussion

**FIGURE 9.**
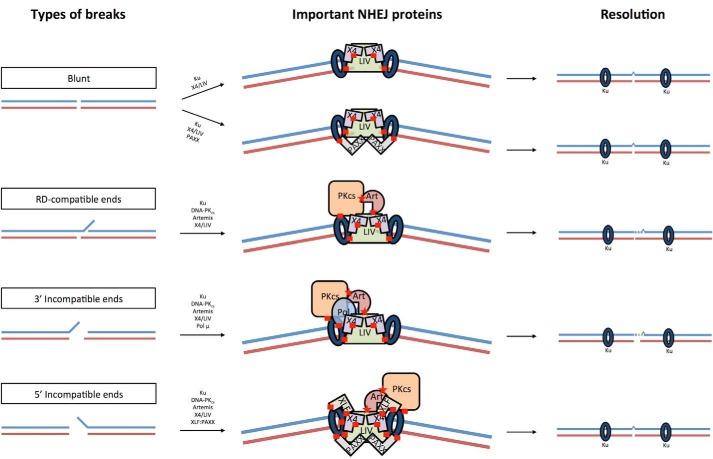
**Diagram of end complex.** This diagram shows how the various NHEJ proteins might associate at the ends. For simplification, we have only depicted the ligation of the top strand, but the bottom strand will also undergo processing. *Red stars* represent interactions that stimulate Artemis activity. *Red squares* represent known protein-protein binding interactions. Ku interacts with LIV at the region containing the two BRCT domains (41). The region between the BRCT domains of LIV interacts with the helical domain of X4 (7–9). The N-terminal head domain of XLF interacts with the N-terminal head domain of X4 (10). The C terminus of PAXX interacts with Ku (11). The N-terminal BRCT domain of Pol μ interacts with the Ku·DNA complex (19). The FAT domain in DNA-PK_cs_ interacts with Ku (42). Artemis is activated by its interaction with DNA-PK_cs_ through its C terminus (amino acids 402- 403). The C terminus of Artemis (amino acids 485–495) interacts with the N-terminal head domain of LIV (20, 21, 31), and the current study indicates that this stimulates Artemis activity (Fig. 2).

Double-stranded DNA breaks arise from ionizing radiation, reactive oxygen species, replication errors, and inadvertent cleavage by nuclear enzymes and by exogenous chemicals. These breaks can create diverse DNA end structures that must be repaired. We have developed a NHEJ reconstitution assay that can be used to compare NHEJ efficiencies and analyze the junctions by analyzing sequences to determine the effects of the proteins involved. It had been previously determined that the ligation of compatible DNA ends is a highly efficient process that requires only the X4·LIV complex and is stimulated by Ku when there is a 1-nt gap to ligate over (18). Thus, we were now interested in determining how complex 5′ and 3′ overhangs were processed and resolved. We find that NHEJ of incompatible DNA ends is less efficient compared with NHEJ of compatible DNA ends. This inefficient process is improved with the addition of Pol μ for 3′ incompatible ends. PAXX alone promotes the NHEJ of blunt ends, whereas PAXX and XLF synergistically enhance NHEJ of a 5′ overhang to a blunt end. We have summarized these observations in the diagram in Fig. 9 (the *red squares* represent known protein interactions, and the *red stars* represent interactions that increase Artemis activity). The conclusions drawn from these experiments support the flexibility and differing NHEJ protein requirements that we previously hypothesized might exist (24).

### 

#### 

##### Ku Contribution to NHEJ

We find that NHEJ of all DNA ends without MH requires Ku. We previously showed that NHEJ of ends with MH does not require Ku (18), and as the ends become more difficult to stabilize, perhaps by transient end annealing, Ku is required. Ku has a high affinity for DNA ends (*K_D_* = 5.9 × 10^−10^ m) (25) and can promote the binding of X4·LIV to the DNA end when it is present (26). This explains why Ku is important for all of the substrates we test here in our study.

##### Artemis·DNA-PK_cs_ Contribution to NHEJ

The Artemis·DNA-PK_cs_ complex is required for hairpin opening of the coding joints in V(D)J recombination (27). Consequently, both Artemis and DNA-PK_cs_-deficient cells fail to cleave this hairpin, consistent with DNA-PK_cs_ activation of Artemis (4). In the context of NHEJ, Artemis-deficient cells are radiosensitive, but the majority of the DSBs in these cells are repaired efficiently (28, 29). Approximately 20% of ionizing radiation-induced DNA DSBs required Artemis for repair (30). Here, we find that Artemis activity is required for NHEJ of DNA substrates with incompatible 5′ and 3′ overhangs. In contrast, blunt-ended DNA undergoes direct ligation. The Artemis·DNA-PK_cs_ complex is able to resect the overhang to reveal regions of MH in RD-compatible ends (Table 1). For DNA ends with incompatible 3′ overhangs, resection occurs, but that is not sufficient for efficient NHEJ because there are no regions of MH (Fig. 5, *A* and *B*). Often, the resection goes deeper into the duplex to allow exposure of other regions of MH (supplemental Table S3). Artemis·DNA-PK_cs_ activity on 5′ overhangs prefers resection of the overhang to result in a blunt DNA end (27). Thus, the 5′ overhang is processed to a blunt-ended substrate, in which direct ligation is favored.

One surprising result was the observation of resection of the 3′ overhangs in Artemis and X4·LIV conditions (Figs. 2, *lane 5*; 3*B*, *lane 4*; and 5, *A*, *lane 5*, and *B*, *lane 5*). It is possible that the reported interaction of Artemis and DNA ligase IV may also activate Artemis activity (20, 21, 31), although to a lesser extent than by DNA-PK_cs_. DNA ligase IV may be able to stimulate Artemis at DNA ends because X4·LIV has an affinity for ligatable DNA ends. (Hairpin DNA ends likely do not bind X4·LIV.)

The dependence of some DSBs on DNA-PK_cs_ and not other DSBs may be relevant to immunoglobulin class switch recombination. During Ig class switch recombination, DNA lesions generated by activation-induced deaminase lead to DSBs. We speculate that after the 5′ nuclease, ExoI, acts, many 3′ overhangs are common, although this may depend on which downstream Ig switch region is being joined to the upstream Sμ DNA end. This would explain why DNA-PK_cs_ is variably required for some Ig switch recombinations but not others (32, 33).

##### Pol μ and λ Contribution to NHEJ

Pol X family members consist of Pol μ, λ, β, and TdT. Pol β is involved in base excision repair and lacks the BRCT domain that is responsible for its interaction with Ku (19). TdT is only expressed in pre-B and pre-T lymphocytes and contributes to increasing immune diversity by adding random nucleotides to coding ends in V(D)J recombination. Thus, TdT has no role in NHEJ outside the context of V(D)J recombination. Pol μ is known to be involved in NHEJ and has the ability to add nucleotides in a template-independent and -dependent manner (18). Pol λ on the other hand, is primarily a template-dependent polymerase. The structural differences in loop 1 of these polymerases have been implicated as the flexible region that promotes template-independent activity (22). Our study shows that Pol μ, but not Pol λ, plays a major role in promoting the NHEJ of 3′ incompatible ends (Fig. 6). Pol μ strongly stimulates NHEJ of 3′ overhangs because nucleotides can be added that can form MH with the 3′ overhang on the other DNA partner (Table 1). The lack of obvious regions of MH in the NHEJ of 3′ incompatible overhangs reduces the ability of Pol λ to find a stable template to initiate synthesis. In contrast, 5′ incompatible ends do not require Pol μ (Fig. 8*C*). The 5′ overhang is resected into a blunt end efficiently by the Artemis·DNA-PK_cs_ complex. This blunt end then undergoes direct ligation as is preferred for blunt ends (Table 1).

*In vivo* studies of POL4 (homolog to mammalian Pol X family members) mutants in yeast have also demonstrated that POL4 is indispensable for 3′ overhangs but not for 5′ overhangs (34). Furthermore, Pol X family members complement POL4 mutants, suggesting that the yeast model is translatable to some aspects of mammalian cells (35). Pol μ has likely evolved to be able to add nucleotides in a template-independent manner to help generate the MH required for ligation. Another study on C57BL/6 MEFs demonstrated that Pol μ was largely responsible for the nucleotide addition in short (1–2 nt) 3′ incompatible ends (36). The observation that overhangs are resected by Artemis·DNA-PK_cs_ followed by the addition of nucleotides by Pol μ supports their importance in increasing junctional diversity during V(D)J recombination.

##### XLF and PAXX Contribution to NHEJ

XLF and PAXX are the most recent NHEJ components discovered, and these interact with XRCC4 and Ku, respectively (10, 11, 37). Single molecule studies have shown that XRCC4/LIV/XLF may assemble at the DNA end with Ku (38). Although XLF-deficient cells are more radiosensitive and biochemical evidence has shown that XLF stimulates NHEJ of incompatible 3′ ends (10, 23), XLF may only be required for the repair of a subset of DNA ends (12). To date, PAXX has been shown to improve blunt end ligation (Fig. 7*B*) (11). We find that XLF and PAXX stimulate NHEJ of a 5′ overhang to a blunt end (Fig. 8*B*). XLF and PAXX do not seem to play a significant role in the NHEJ of substrates with 3′ overhangs. The role of XLF and PAXX may thus be to stabilize ends that do not utilize MH for ligation.

##### Concluding Comments

As summarized in Table 2, some DNA end configurations may be ligatable with the involvement of a small number of NHEJ proteins and others require more NHEJ proteins. Our biochemical reconstitution supports this view, which is consistent with *in vivo* studies.

## Experimental Procedures

### 

#### 

##### Oligonucleotides and DNA Substrates

Oligonucleotides used in this study were synthesized by Integrated DNA Technologies, Inc. (San Diego, CA). They were purified and 5′ end-radiolabeled with [γ-^32^P]ATP (3,000 Ci/mmol) (PerkinElmer Life Sciences) or 3′ end-radiolabeled using [α-^32^P]dNTP (3,000 Ci/mmol) (PerkinElmer Life Sciences). The oligonucleotides were labeled and purified as previously described (15). Oligonucleotides were quantified by using a UV spectrophotometer to determine the absorbance at 280 nm. The concentration of dsDNA was determined by annealing known concentrations of both oligonucleotides. For unlabeled DNA, equal amounts of each oligonucleotide were annealed. For radiolabeled dsDNA, a 20% excess of unlabeled oligonucleotide was added to the radiolabeled oligonucleotide to ensure that all labeled substrates were in duplex conformation.

The sequences of the oligonucleotides used in this study are as follows: HC101, 5′-C*G*T* T*AA GTA TCT GCA TCT TAC TTG ATG GAG GAT CCT GTC ACG TGC TAG ACT ACT GGT CAA GCG CAT CGA GAA CCC CCC TTT TTT-3′; HC102, 5′-GGT TCT CGA TGC GCT TGA CCA GTA GTC TAG CAC GTG ACA GGA TCC TCC ATC AAG TAA GAT GCA GAT ACT TAA CG-Biotin-3′; HC105, 5′-CTA GAC TAC TGG TCA AGC-3′; HC114, 5′-TGT ACA TAT ATC AGT GTC TGC-3′; HC115, 5′-GAT GCC TCC AAG GTC GAC GAT GCA GAC ACT GAT ATA TGT ACA GAT TCG GTT GAT CAT AGC ACA ATG CCT GCT GAA CCC ACT ATC G-3′; HC116, 5′-Biotin-CGA TAG TGG GTT CAG CAG GCA TTG TGC TAT GAT CAA CCG AAT CTG TAC ATA TAT CAG TGT CTG CAT CGT CGA CCT TGG AGG CAT CGG GG-3′; HC119, 5′-Biotin-CGA TAG TGG GTT CAG CAG GCA TTG TGC TAT GAT CAA CCG AAT CTG TAC ATA TAT CAG TGT CTG CAT CGT CGA CCT TGG AGG CAT CTT TT-3′; HC120, 5′-Biotin-CGA TAG TGG GTT CAG CAG GCA TTG TGC TAT GAT CAA CCG AAT CTG TAC ATA TAT CAG TGT CTG CAT CGT CGA CCT TGG AGG CAT C-3′; HC121, 5′-C*G*T* T*AA GTA TCT GCA TCT TAC TTG ATG GAG GAT CCT GTC ACG TGC TAG ACT ACT GGT CAA GCG CAT CGA GAA CC-3′; HC123, 5′-Biotin-CGA TAG TGG GTT CAG CAG GCA TTG TGC TAT GAT CAA CCG AAT CTG TAC ATA TAT CAG TGT CTG CAT CGT CGA CCT TGG AGG CAT CGG-3′; HC124, 5′-Biotin-CGA TAG TGG GTT CAG CAG GCA TTG TGC TAT GAT CAA CCG AAT CTG TAC ATA TAT CAG TGT CTG CAT CGT CGA CCT TGG AGG CAT CG-3′; JG187, 5′-TGC TAG ACT ACT GGT CAA GC-3′; JG188, 5′-TGC ATC CGT CAA GTA AGA TG-3′; JG226, 5′-ACG AGC CCG ATC CGC TTG ACC AGT AGT CTA GCA CGT GAC GAT TGC ATC CGT CAA GTA AGA TGC AGA TAC TTA AC-3′; and JG277, 5′-TTT TTT CCC CGT TAA GTA TCT GCA TCT TAC TTG ACG GAT GCA ATC GTC ACG TGC TAG ACT ACT GGT CAA GCG GAT CGG GCT CG-ddG-3′. (The asterisks represent phosphorothioate bonds, and ddG represents a dideoxyguanidine.)

##### Protein Expression and Purification

Purification of Ku, DNA-PK_cs_, and Artemis was done as previously described (15). Ku70-His/Ku80 was purified from Hi-Five insect cells (Invitrogen, B855-02) by Ni-NTA affinity chromatography, dsDNA(oligo) affinity chromatography, and anion exchange chromatography. Endogenous DNA-PK_cs_ was purified from HeLa cells using a series of anion exchange, cation exchange, dsDNA(oligo) affinity, and size exclusion chromatography. Artemis-his was purified from Sf9 insect cells (Life Technologies, 11496-015) by Ni-NTA affinity chromatography and anion exchange chromatography. Native Pol μ and Pol λ were purified as described previously (18). The X4·LIV complex was purified as described (39). Briefly, X4·LIV was purified from Hi-Five insect cells by Ni-NTA affinity chromatography, anion exchange, size exclusion, and cation exchange chromatography. XLF-myc-His protein was purified from 293T cells by Ni-NTA affinity and anion exchange chromatography as described (40). PAXX was purified from a pHAT4 vector encoding for His-PAXX in BL21(DE3) *Escherichia coli* using Ni-NTA affinity chromatography, followed by TEV protease-mediated cleavage of the N-terminal his-tag, and size exclusion chromatography as described (11).

##### NHEJ Assay

*In vitro* NHEJ assays were performed in a volume of 10 μl with a buffer composition of 25 mm Tris-HCl (pH 8.0), 75 mm KCl, 10 mm MgCl_2_, and 1 mm DTT, 10% PEG 8000. The reactions consisted of 20 nm of ^32^P-labeled DNA and 20 nm of unlabeled DNA substrate incubated with 0.5 mm ATP and 200 nm streptavidin (in reactions with biotinylated substrates) at 37 °C for 60 min, unless specified otherwise. In addition, 100 μm dNTPs were added to reactions containing a polymerase. The streptavidin is used to block the biotinylated end of the DNA from proteins. Phenol-chloroform extraction was immediately performed on the reactions to deproteinize the samples. The reactions were then analyzed on an 8% denaturing PAGE. The gels were then dried and exposed to a PhosphorImager screen overnight. The screen was scanned, and quantification was performed in Quantity One® one-dimensional analysis (Bio-Rad) by dividing the NHEJ product band intensity by the total signal in each lane.

##### Junction Sequence Analysis

The dried denaturing PAGE gels were exposed to X-ray film for at least 24 h. The developed film was then overlaid on the gel to cut out individual NHEJ ligation bands. The bands were incubated in 100 μl of 10 mm Tris (pH8) - 1 mm EDTA at 37 °C overnight to allow ample time for DNA diffusion. PCR was performed on the samples using primers (HC105 and HC114 for 5′-labeled substrates and JG187 and JG188 for 3′-labeled substrates) that flank the junction. The PCR product was then TA-cloned into a pGEM-T Easy vector by following the product manual (catalog no. TM042, Promega). TA-cloned product was diluted 2-fold in ddH_2_O and transformed into DH10B competent cells, and transformants were plated on LB/Amp/X-gal agar plates. White colonies were selected for plasmid mini-prep and Sanger sequencing.

## Author Contributions

H. H. Y. C. performed nearly all of the experiments, whereas C. A. G. helped and performed a subset. G. W. purified DNA-PK_cs_. T. O., T. L. B., and S. P. J. provided key reagents and advice on PAXX purification. M. R. L. conceived the study. H. H. Y. C. and M. R. L. designed the experiments and wrote the manuscript with input from all authors.

## Supplementary Material

Supplemental Data

## Footnotes

3 The abbreviations used are:

DSB: double-stranded break
NHEJ: nonhomologous DNA end joining
DNA-PK_cs_: DNA-dependent protein kinase catalytic subunit
nt: nucleotide(s)
MH: microhomology
RD: resection-dependent
TdT: terminal deoxynucleotidyl transferase
Ni-NTA: nickel-nitrilotriacetic acid.

## References

[1] Martin G. M., Smith A. C., Ketterer D. J., Ogburn C. E., and Disteche C. M. (1985) Increased chromosomal aberrations in first metaphases of cells isolated from the kidneys of aged mice. Israel J. Med. Sci. 21, 296–301 3997491

[2] Rich T., Allen R. L., and Wyllie A. H. (2000) Defying death after DNA damage. Nature 407, 777–783 11048728 10.1038/35037717

[3] Lieber M. R., and Karanjawala Z. E. (2004) Ageing, repetitive genomes and DNA damage. Nat. Rev. Mol. Cell Biol. 5, 69–75 14708011 10.1038/nrm1281

[4] Lieber M. R. (2010) The mechanism of double-strand DNA break repair by the nonhomologous DNA end-joining pathway. Annu. Rev. Biochem. 79, 181–211 20192759 10.1146/annurev.biochem.052308.093131 PMC3079308

[5] Jackson S. P., and Bartek J. (2009) The DNA-damage response in human biology and disease. Nature 461, 1071–1078 19847258 10.1038/nature08467 PMC2906700

[6] Downs J. A., and Jackson S. P. (2004) A means to a DNA end: the many roles of Ku. Nat. Rev. Mol. Cell Biol. 5, 367–378 15122350 10.1038/nrm1367

[7] Sibanda B. L., Critchlow S. E., Begun J., Pei X. Y., Jackson S. P., Blundell T. L., and Pellegrini L. (2001) Crystal structure of an Xrcc4-DNA ligase IV complex. Nat. Struct. Biol 8, 1015–1019 11702069 10.1038/nsb725

[8] Grawunder U., Zimmer D., Kulesza P., and Lieber M. R. (1998) Requirement for an interaction of XRCC4 with DNA ligase IV for wild-type V(D)J recombination and DNA double-strand break repair in vivo. J. Biol. Chem. 273, 24708–24714 9733770 10.1074/jbc.273.38.24708

[9] Grawunder U., Zimmer D., and Lieber M. R. (1998) DNA ligase IV binds to XRCC4 via a motif located between rather than within its BRCT domains. Curr. Biol. 8, 873–876 9705934 10.1016/s0960-9822(07)00349-1

[10] Ahnesorg P., Smith P., and Jackson S. P. (2006) XLF interacts with the XRCC4-DNA ligase IV complex to promote nonhomologous end-joining. Cell 124, 301–313 16439205 10.1016/j.cell.2005.12.031

[11] Ochi T., Blackford A. N., Coates J., Jhujh S., Mehmood S., Tamura N., Travers J., Wu Q., Draviam V. M., Robinson C. V., Blundell T. L., and Jackson S. P. (2015) DNA repair: PAXX, a paralog of XRCC4 and XLF, interacts with Ku to promote DNA double-strand break repair. Science 347, 185–188 25574025 10.1126/science.1261971 PMC4338599

[12] Roy S., de Melo A. J., Xu Y., Tadi S. K., Négrel A., Hendrickson E., Modesti M., and Meek K. (2015) XRCC4/XLF interaction is variably required for DNA repair and is not required for ligase IV stimulation. Mol. Cell. Biol. 35, 3017–3028 26100018 10.1128/MCB.01503-14 PMC4525314

[13] Buck D., Malivert L., de Chasseval R., Barraud A., Fondanèche M.-C., Sanal O., Plebani A., Stéphan J.-L., Hufnagel M., le Deist F., Fischer A., Durandy A., de Villartay J.-P., and Revy P. (2006) Cernunnos, a novel nonhomologous end-joining factor, is mutated in human immunodeficiency with microcephaly. Cell 124, 287–299 16439204 10.1016/j.cell.2005.12.030

[14] Xing M., Yang M., Huo W., Feng F., Wei L., Jiang W., Ning S., Yan Z., Li W., Wang Q., Hou M., Dong C., Guo R., Gao G., Ji J., Zha S., Lan L., Liang H., and Xu D. (2015) Interactome analysis identifies a new paralogue of XRCC4 in non-homologous end joining DNA repair pathway. Nat. Commun. 6, 6233 25670504 10.1038/ncomms7233 PMC4339890

[15] Chang H. H., Watanabe G., and Lieber M. R. (2015) Unifying the DNA end-processing roles of the Artemis nuclease: Ku-dependent artemis resection at blunt DNA ends. J. Biol. Chem. 290, 24036–24050 26276388 10.1074/jbc.M115.680900 PMC4591795

[16] Li S., Kanno S., Watanabe R., Ogiwara H., Kohno T., Watanabe G., Yasui A., and Lieber M. R. (2011) Polynucleotide kinase and aprataxin-like forkhead-associated protein (PALF) acts as both a single-stranded DNA endonuclease and a single-stranded DNA 3′ exonuclease and can participate in DNA end joining in a biochemical system. J. Biol. Chem. 286, 36368–36377 21885877 10.1074/jbc.M111.287797 PMC3196146

[17] Li S., Chang H. H., Niewolik D., Hedrick M. P., Pinkerton A. B., Hassig C. A., Schwarz K., and Lieber M. R. (2014) Evidence that the DNA endonuclease ARTEMIS also has intrinsic 5′-exonuclease activity. J. Biol. Chem. 289, 7825–7834 24500713 10.1074/jbc.M113.544874 PMC3953294

[18] Gu J., Lu H., Tippin B., Shimazaki N., Goodman M. F., and Lieber M. R. (2007) XRCC4:DNA ligase IV can ligate incompatible DNA ends and can ligate across gaps. EMBO J. 26, 1010–1023 17290226 10.1038/sj.emboj.7601559 PMC1852838

[19] Ma Y., Lu H., Tippin B., Goodman M. F., Shimazaki N., Koiwai O., Hsieh C.-L., Schwarz K., and Lieber M. R. (2004) A biochemically defined system for mammalian nonhomologous DNA end joining. Mol. Cell 16, 701–713 15574326 10.1016/j.molcel.2004.11.017

[20] Malu S., De Ioannes P., Kozlov M., Greene M., Francis D., Hanna M., Pena J., Escalante C. R., Kurosawa A., Erdjument-Bromage H., Tempst P., Adachi N., Vezzoni P., Villa A., Aggarwal A. K., et al (2012) Artemis C-terminal region facilitates V(D)J recombination through its interactions with DNA Ligase IV and DNA-PKcs. J. Exp. Med. 209, 955–963 22529269 10.1084/jem.20111437 PMC3348108

[21] De Ioannes P., Malu S., Cortes P., and Aggarwal A. K. (2012) Structural basis of DNA ligase IV-Artemis interaction in nonhomologous end-joining. Cell Rep 2, 1505–1512 23219551 10.1016/j.celrep.2012.11.004 PMC3538150

[22] Moon A. F., Garcia-Diaz M., Bebenek K., Davis B. J., Zhong X., Ramsden D. A., Kunkel T. A., and Pedersen L. C. (2007) Structural insight into the substrate specificity of DNA polymerase mu. Nat. Struct. Mol. Biol. 14, 45–53 17159995 10.1038/nsmb1180

[23] Gu J., Lu H., Tsai A. G., Schwarz K., and Lieber M. R. (2007) Single-stranded DNA ligation and XLF-stimulated incompatible DNA end ligation by the XRCC4-DNA ligase IV complex: influence of terminal DNA sequence. Nucleic Acids Res. 35, 5755–5762 17717001 10.1093/nar/gkm579 PMC2034460

[24] Pannunzio N. R., Li S., Watanabe G., and Lieber M. R. (2014) Nonhomologous end joining often uses microhomology: implications for alternative end joining. DNA Repair 17, 74–80 24613510 10.1016/j.dnarep.2014.02.006 PMC4440676

[25] Mimori T., and Hardin J. A. (1986) Mechanism of interaction between Ku protein and DNA. J. Biol. Chem. 261, 10375–10379 3015926

[26] Nick McElhinny S. A., Snowden C. M., McCarville J., and Ramsden D. A. (2000) Ku recruits the XRCC4-ligase IV complex to DNA ends. Mol. Cell. Biol. 20, 2996–3003 10757784 10.1128/mcb.20.9.2996-3003.2000 PMC85565

[27] Ma Y., Pannicke U., Schwarz K., and Lieber M. R. (2002) Hairpin opening and overhang processing by an Artemis·DNA-PKcs complex in V(D)J recombination and in nonhomologous end joining. Cell 108, 781–794 11955432 10.1016/s0092-8674(02)00671-2

[28] Nicolas N., Moshous D., Cavazzana-Calvo M., Papadopoulo D., de Chasseval R., Le Deist F., Fischer A., and de Villartay J.-P. (1998) A human severe combined immunodeficiency condition with increased sensitivity to ionizing radiation and impaired V(D)J rearrangements defines a new DNA reccombination/repair deficiency. J. Exp. Med. 188, 627–634 9705945 10.1084/jem.188.4.627 PMC2213354

[29] Moshous D., Callebaut I., de Chasseval R., Corneo B., Cavazzana-Calvo M., Le Deist F., Tezcan I., Sanal O., Bertrand Y., Philippe N., Fischer A., and de Villartay J.-P. (2001) Artemis, a novel DNA double-strand break repair/V(D)J recombination protein, is mutated in human severe combined immune deficiency. Cell 105, 177–186 11336668 10.1016/s0092-8674(01)00309-9

[30] Riballo E., Kühne M., Rief N., Doherty A., Smith G. C., Recio M.-J., Reis C., Dahm K., Fricke A., Krempler A., Parker A. R., Jackson S. P., Gennery A., Jeggo P. A., and Löbrich M. (2004) A pathway of double-strand break rejoining dependent upon ATM, Artemis, and proteins locating to gamma-H2AX foci. Mol. Cell 16, 715–724 15574327 10.1016/j.molcel.2004.10.029

[31] Ochi T., Gu X., and Blundell T. L. (2013) Structure of the catalytic region of DNA ligase IV in complex with an Artemis fragment sheds light on double-strand break repair. Structure 21, 672–679 23523427 10.1016/j.str.2013.02.014 PMC3664939

[32] Bosma G. C., Kim J., Urich T., Fath D. M., Cotticelli M. G., Ruetsch N. R., Radic M. Z., and Bosma M. J. (2002) DNA-dependent protein kinase activity is not required for immunoglobulin class switching. J. Exp. Med. 196, 1483–1495 12461083 10.1084/jem.20001871 PMC2194268

[33] Franco S., Murphy M. M., Li G., Borjeson T., Boboila C., and Alt F. W. (2008) DNA-PKcs and Artemis function in the end-joining phase of immunoglobulin heavy chain class switch recombination. J. Exp. Med. 205, 557–564 18316419 10.1084/jem.20080044 PMC2275379

[34] Liang Z., Sunder S., Nallasivam S., and Wilson T. E. (2016) Overhang polarity of chromosomal double-strand breaks impacts kinetics and fidelity of yeast non-homologous end joining. Nucleic Acids Res. 44, 2769–2781 26773053 10.1093/nar/gkw013 PMC4824102

[35] Daley J. M., and Wilson T. E. (2005) Rejoining of DNA double-strand breaks as a function of overhang length. Mol. Cell. Biol. 25, 896–906 15657419 10.1128/MCB.25.3.896-906.2005 PMC544009

[36] Pryor J. M., Waters C. A., Aza A., Asagoshi K., Strom C., Mieczkowski P. A., Blanco L., and Ramsden D. A. (2015) Essential role for polymerase specialization in cellular nonhomologous end joining. Proc. Natl. Acad. Sci. U.S.A. 112, E4537–4545 26240371 10.1073/pnas.1505805112 PMC4547266

[37] Dai Y., Kysela B., Hanakahi L. A., Manolis K., Riballo E., Stumm M., Harville T. O., West S. C., Oettinger M. A., and Jeggo P. A. (2003) Nonhomologous end joining and V(D)J recombination require an additional factor. Proc. Natl. Acad. Sci. 100, 2462–2467 12604777 10.1073/pnas.0437964100 PMC151363

[38] Reid D. A., Keegan S., Leo-Macias A., Watanabe G., Strande N. T., Chang H. H., Oksuz B. A., Fenyo D., Lieber M. R., Ramsden D. A., and Rothenberg E. (2015) Organization and dynamics of the nonhomologous end-joining machinery during DNA double-strand break repair. Proc. Natl. Acad. Sci. U.S.A. 112, E2575–E2584 25941401 10.1073/pnas.1420115112 PMC4443322

[39] Chen X., Pascal J., Vijayakumar S., Wilson G. M., Ellenberger T., and Tomkinson A. E. (2006) Human DNA ligases I, III, and IV-purification and new specific assays for these enzymes. Methods Enzymol. 409, 39–52 16793394 10.1016/S0076-6879(05)09003-8

[40] Lu H., Pannicke U., Schwarz K., and Lieber M. R. (2007) Length-dependent binding of human XLF to DNA and stimulation of XRCC4.DNA ligase IV activity. J. Biol. Chem. 282, 11155–11162 17317666 10.1074/jbc.M609904200

[41] Costantini S., Woodbine L., Andreoli L., Jeggo P. A., and Vindigni A. (2007) Interaction of the Ku heterodimer with the DNA ligase IV/Xrcc4 complex and its regulation by DNA-PK. DNA Repair 6, 712–722 17241822 10.1016/j.dnarep.2006.12.007

[42] Spagnolo L., Rivera-Calzada A., Pearl L. H., and Llorca O. (2006) Three-dimensional structure of the human DNA-PKcs/Ku70/Ku80 complex assembled on DNA and its implications for DNA DSB repair. Mol. Cell 22, 511–519 16713581 10.1016/j.molcel.2006.04.013

