# Supplementary material for: Different DNA End Configurations Dictate Which NHEJ Components Are Most Important for Joining Efficiency
Source: J Biol Chem. 2016 Oct 4;291(47):24377–89. doi: 10.1074/jbc.M116.752329 (PMC5114395; doi:10.1074/jbc.M116.752329)
Supplement: Supplemental Data [file supp_291_47_24377__index.html]

Different DNA End Configurations Dictate Which NHEJ Components are Most Important for Joining Efficiency — Different DNA End Configurations Dictate Which NHEJ Components are Most Important for Joining Efficiency — Different DNA End Configurations Dictate Which NHEJ Components Are Most Important for Joining Efficiency — DNA Ends Dictate the NHEJ Components Required for Joining — Supplemental Data 

# Different DNA End Configurations Dictate Which NHEJ Components Are Most Important for Joining Efficiency

## Supplemental Data

- Supplement to be published online (.pdf, 147 KB) - Supplement to be published online
