# Supplementary material for: Different DNA End Configurations Dictate Which NHEJ Components Are Most Important for Joining Efficiency
Source: J Biol Chem. 2016 Oct 4;291(47):24377–89. doi: 10.1074/jbc.M116.752329 (PMC5114395; doi:10.1074/jbc.M116.752329)
Supplement: Supplemental Data [file 10.1074_M116.752329_jbc.M116.752329-1.pdf]

RD-compatible ends

GCGCATCGAGAACC**CCCCTTTTTT**	GATGCCTCCAAGG
CGCGTAGCTCTTGG	**GGGG**CTACGGAGGTTCC

Proteins	Junctions	Junctions	MH	n	NHEJ
Artemis	GCGCATCGAGAACC**CCC**-------	GATGCCTCCAAGG	3	2	+
	GCGCATCGAGAACC**CCCC**------	GATGCCTCCAAGG	4	18	
Ku	GCGCATCGAGAACC**CC**--------	GATGCCTCCCAGG	2	2	+
Artemis	GCGCATCGAGAACC**CCC**-------	GATGCCTCCAAGG	3	5	
	GCGCATCGAGAACC**CCCC**------	GATGCCTCCAAGG	4	9	
DNA-PKcs	GCGCATCGAGAACC**CCC**-------	GATGCCTCCAAGG	3	10	++
Artemis	GCGCATCGAGAACC**CCCC**------	GATGCCTCCAAGG	4	9	
	GCGCATCGAGAACC**CCCC**------	-ATGCCTCCAAGG	4	1	
Ku	GCGCATCGAGAACC**CC**--------	GATGCCTCCAAGG	2	1	+++++
DNA-PKcs	GCGCATCGAGAACC**CCC**-------	GATGCCTCCAAGG	3	8	
Artemis	GCGCATCGAGAACC**CCCC**------	GATGCCTCCAAGG	4	7	
	GCGCATCGAGAACC**CCCCTTTT**--	---GCCTCCAAGG	1	1	
Ku	GCGCATCGAGAACC**CCC**-------	GATGCCTCCAAGG	3	2	+++++
DNA-PKcs	GCGCATCGAGAACC**CCCC**------	GATGCCTCCAAGG	4	5	
Artemis	GCGCATCGAGAACC**CCCCT**-----	GATGCCTCCAAGG	4	1	
Pol μ					
Ku	GCGCATCGAGAACC**CC**--------	GATGCCTCCAAGG	2	1	+++++
DNA-PKcs	GCGCATCGAGAACC**CCC**-------	GATGCCTCCAAGG	3	2	
Artemis	GCGCATCGAGAACC**CCCC**------	GATGCCTCCAAGG	4	5	
Pol λ					

Table S1. Sequence results of RD-compatible ends. Sequencing results of the NHEJ junctions with a column for proteins included, sequenced junctions, microhomology (MH) utilized at the junction, the number of molecules sequenced (n), and the relative NHEJ efficiencies (NHEJ). All conditions include X4/LIV. Dashes (-) represent resected bases. Only the top strand is shown.

3’ incompatible ends

GCGCATCGAGAACC**CCCCTTTTTT**	GATGCCTCCAAGG
CGCGTAGCTCTTGG	**TTTT**CTACGGAGGTTCC

Proteins	Junctions		Junctions	MH	n	NHEJ
Ku	GCGCATCG----------------	**aaaa**	GATGCCTCCAAGG	1	2	
Artemis	GCGCATCGAGAA------------		GATGCCTCCAAGG	2	2	
	GCGCATCGAGAA------------	**aa**	GATGCCTCCAAGG	2	1	
	GCGCATCGAGAACC----------		GATGCCTCCAAGG	2	2	+
	GCGCATCGAGAACC----------		--TcCCTCCAAGG	0	1	
	GCGCATCGAGAACC**C**---------		----CCTCCAAGG	2	1	
	GCGCATCGAGAACC**CCC**-------		------TCCAAGG	2	1	
DNA-PKcs	GCGCAT------------------		GATGCCTCCAAGG	0	3	
Artemis	GCGCATCGAGAA------------		--TGCCTCCAAGG	1	3	+
	GCGCATCGAGAACC**CCC**-------		------TCCAAGG	2	2	
	GCGCATCGAGAACC**CCCCT**-----		-------CCAAGG	3	1	
Ku	GCGCATCGA---------------	**aaaaa**	GATGCCTCCAAGG	0	1	
DNA-PKcs	GCGCATCGAGAA------------		GATGCCTCCAAGG	2	2	
Artemis	GCGCATCGAGAA------------	**aa**	GATGCCTCCAAGG	2	6	+
	GCGCATCGAGAAC-----------		GATGCCTCCAAGG	2	1	
Ku	GCGCATCGAGAA------------		GATGCCTCCAAGG	2	2	
DNA-PKcs	GCGCATCGAGAA------------	**aagg**	--TGCCTCCAAGG	3+1	1	
Artemis	GCGCATCGAGAACC**CC**--------	**acaaaa**	GATGCCTCCAAGG	4	1	
Pol µ	GCGCATCGAGAACC**CCC**-------	**aaaa**	GATGCCTCCAAGG	4	1	
	GCGCATCGAGAACC**CCCC**------	**aaaa**	GATGCCTCCAAGG	4	3	+++
	GCGCATCGAGAACC**CCCCT**-----	**g**	GATGCCTCCAAGG	0	1	
	GCGCATCGAGAACC**CCCCT**-----	**aa**	GATGCCTCCAAGG	2	4	
	GCGCATCGAGAACC**CCCCT**-----	**aaa**	GATGCCTCCAAGG	3	1	
	GCGCATCGAGAACC**CCCCTTTT**--	**aaaaa**	GATGCCTCCAAGG	4	1	
Ku	GCGCATCGA---------------	**aa**	GATGCCTCCAAGG	3	1	
DNA-PKcs	GCGCATCGA---------------	**aaa**	GATGCCTCCAAGG	4	1	
Artemis	GCGCATCGAGAA------------	**aa**	GATGCCTCCAAGG	4	2	+
Pol λ	GCGCATCGAGAACC----------		---------AAGG	2	1	
	GCGCATCGAGAACC**CC**--------		----CCTCCAAGG	0	1	
Ku	GCGCATCGA---------------	**aaa**	GATGCCTCCAAGG	4	1	
DNA-PKcs	GCGCATCGAGAACC**CCC**-------	**aaaa**	GATGCCTCCAAGG	4	3	
Artemis	GCGCATCGAGAACC**CCCC**------		GATGCCTCCAAGG	0	1	+++
XLF	GCGCATCGAGAACC**CCCC**------	**cccaaa**	GATGCCTCCAAGG	3	1	
PAXX	GCGCATCGAGAACC**CCCCT**-----	**aa**	GATGCCTCCAAGG	2	6	
Pol µ						

Table S2. Sequence results of Incompatible 3’ ends. Sequencing results of the NHEJ junctions with a column for proteins included, sequenced junctions, microhomology (MH) utilized at the junction, the number of molecules sequenced (n), and the relative NHEJ efficiencies (NHEJ). All conditions include X4/LIV. Dashes (-) represent resected bases, and bolded lowercase letters represent added bases. Only the top strand is shown.

3’ ovh + blunt

GCGCATCGAGAACC**CCCCTTTTTT**	GATGCCTCCAAGG
CGCGTAGCTCTTGG	CTACGGAGGTTCC

Proteins	Junctions		Junctions	MH	n	NHEJ
Ku Artemis	GCGCAT------------------		---GCCTCCAAGG	2	4	+
	GCGCATCGAGAAC-----------		GATGCCTCCAAGG	0	1	
	GCGCATCGAGAACC**CC**--------		------TCCAAGG	2	1	
	GCGCATCGAGAACC**CCC**-------		------TCCAAGG	2	1	
	GCGCATCGAGAACC**CCCCT**-----		-------CCAAGG	3	2	
DNA-PKcs Artemis	GCGCAT------------------		---GCCTCCAAGG	2	2	+
	GCGCATCGAGAA------------		-----------GG	2	1	
	GCGCATCGAGAA------------		GATGCCTCCAAGG	0	1	
	GCGCATCGAGAAC-----------		GATGCCTCCAAGG	0	1	
	GCGCATCGAGAACC----------		GATGCCTCCAAGG	0	1	
	GCGCATCGAGAACC**CCC**-------		------TCCAAGG	2	1	
	GCGCATCGAGAACC**CCCC**------		-----CTCCAAGG	1	1	
Ku DNA-PKcs Artemis	GCGCAT------------------		---GCCTCCAAGG	2	2	+
	GCGCATCGAGAAC-----------		------------G	0	1	
	GCGCATCGAGAAC-----------		---GCCTCCAAGG	0	1	
	GCGCATCGAGAAC-----------		GATGCCTCCAAGG	0	2	
	GCGCATCGAGAACC----------		GATGCCTCCAAGG	0	3	
	GCGCATCGAGAACC**C**---------		GATGCCTCCAAGG	0	1	
	GCGCATCGAGAACC**CCC**-------		------TCCAAGG	2	1	
Ku DNA-PKcs Artemis Pol μ	GCGCATCGAGAACC**C**---------		GATGCCTCCAAGG	0	1	+++
	GCGCATCGAGAACC**C**---------	**ag**	-----------GG	3+1	1	
	GCGCATCGAGAACC**CCC**-------	**a**	GATGCCTCCAAGG	0	1	
	GCGCATCGAGAACC**CCCC**------		GATGCCTCCAAGG	0	9	
	GCGCATCGAGAACC**CCCCTT**----		-----------GG	0	1	
Ku DNA-PKcs Artemis Pol λ	GCGCATCGAG--------------		GATGCCTCCAAGG	0	1	+
	GCGCATCGAGAACC**C**---------		---------AAGG	2	1	
	GCGCATCGAGAACC**C**---------		------TCCAAGG	2	1	
	GCGCATCGAGAACC**CCC**-------		------TCCAAGG	2	1	
	GCGCATCGAGAACC**CCCCT**-----		-------CCAAGG	3	1	
	GCGCATCGAGAACC**CCCCT**-----		---GCCTCCAAGG	1	1	
Ku DNA-PKcs Artemis XLF PAXX Pol μ	GC----------------------		GATGCCTCCAAGG	1	1	++
	GCGCATCGAGAACC**C**---------		---GCCTCCAAGG	0	1	
	GCGCATCGAGAACC**CC**--------	**ac**	GATGCCTCCAAGG	0	1	
	GCGCATCGAGAACC**CC**--------	**act**	GATGCCTCCAAGG	0	1	
	GCGCATCGAGAACC**CCC**-------		GATGCCTCCAAGG	0	2	
	GCGCATCGAGAACC**CCCC**------		-ATGCCTCCAAGG	0	1	
	GCGCATCGAGAACC**CCCC**------		GATGCCTCCAAGG	0	5	
	GCGCATCGAGAACC**CCCC**------	**c**	-ATGCCTCCAAGG	0	1	
	GCGCATCGAGAACC**CCCC**------	**c**	GATGCCTCCAAGG	0	2	
	GCGCATCGAGAACC**CCCTTT**----		---GCCTCCAAGG	1	1	

Table S3. Sequence results of 3’ overhang with blunt-ended DNA. Sequencing results of the NHEJ junctions with a column for proteins included, sequenced junctions, microhomology (MH) utilized at the junction, the number of molecules sequenced (n), and the relative NHEJ efficiencies (NHEJ). All conditions include X4/LIV. Dashes (-) represent resected bases, and bolded lowercase letters represent added bases. Only the top strand is shown.

blunt + blunt

GCGCATCGAGAACC	GATGCCTCCAAGG
CGCGTAGCTCTTGG	CTACGGAGGTTCC

	Junctions	Junctions	MH	n	NHEJ
Ku	GCGCATCGAGAACC	GATGCCTCCAAGG	0	12	++++
	GCG_ATCGAGAACC	GATGCCTCCAAGG	0	1	
	GCGCATCG_GAACC	GATGCCTCCAAGG	0	1	
Ku<br>DNA-PKcs	GCGCATCGAGAACC	GATGCCTCCAAGG	0	8	++
Ku<br>Artemis	GCGCATCGAGAACC	-ATGCCTCCAAGG	1	1	++++
	GCGCATCGAGAACC	GATGCCTCCAAGG	0	4	
	GCG_ATCGAGAACC	GATGCCTCCAAGG	0	1	
Ku<br>DNA-PKcs<br>Artemis	GCGCATCGAGAACC	GATGCCTCCAAGG	0	3	++
Ku<br>DNA-PKcs<br>Artemis<br>XLF<br>PAXX	GCGCATCGAGAACC	GATGCCTCCAAGG	0	6	++++

Table S4. Sequence results of Blunt-ended DNA. Sequencing results of the NHEJ junctions with a column for proteins included, sequenced junctions, microhomology (MH) utilized at the junction, the number of molecules sequenced (n), and the relative NHEJ efficiencies (NHEJ). All conditions include X4/LIV. Dashes (-) represent resected bases, and underscores (_) represent deletions. Only the top strand is shown.

5’ overhang + blunt

CGGATCGGGCTCGT	**TTTTTTCCC**CGTTAAGTATCTG	
GCCTAGCCCGAGCA	GCAATTCATAGAC	**NHEJ**

**Proteins**	**Junctions**	**Junctions**	**MH**	**n**	**NHEJ**
Ku<br>Artemis<br>XLF<br>PAXX	CGGATCGGGCT---<br>CGGATCGGGCTCGT<br>CGGATCGGGCTCGT	---------CGTTAAGTATCTG<br>**---------**CGTTAAGTATCTG<br>**TTTTTTCCC**CGTTAAGTATCTG	0<br>0<br>0	1<br>4<br>1	**+**
Ku<br>DNA-PKcs<br>Artemis<br>XLF<br>PAXX	CGGATCGGGCTCGT<br>CGGATCGGGCTCGT	---------CGTTAAGTATCTG<br>**TTTTTTCCC**CGTTAAGTATCTG	0<br>0	8<br>1	**++**

Table S5. Sequence results of 5’ overhang with blunt-ended DNA. Sequencing results of the NHEJ junctions with a column for proteins included, sequenced junctions, microhomology (MH) utilized at the junction, the number of molecules sequenced (n), and the relative NHEJ efficiencies (NHEJ). All conditions include X4/LIV. Dashes (-) represent resected bases. Only the top strand is shown.
